# Repetition of the Dose in the Treatment

**Published:** 1885-05

**Authors:** Julius Schmitt

**Affiliations:** Rochester, N. Y.


### CLINICAL BUREAU.

---

### REPETITION OF THE DOSE IN THE TREATMENT.

JULIUS SCHMITT, M. D., ROCHESTER, N. Y.

[Read before the Central New York Homœopathic Medical Society.]

We read in Hahnemann's *Chronic Diseases*, page 155, second German edition of 1835, as follows: "Chief rule in the treatment of chronic diseases remains to let the dose of the suitable homœopathic remedy, carefully selected according to the symptoms of the sick, act undisturbed as long as the cure is visibly advanced and the amelioration so progresses apparently—a fact which forbids any new ordination or any interruption by another medicine, and also the immediate repetition of the same remedy."

How often has this golden rule been neglected! How many cases of curable chronic diseases have been made incurable by this neglect! How often have the old, experienced followers of our immortal master warned against pernicious haste in repeating or changing remedies! And in spite of all this it seems to me that the younger practitioner cannot curb his anxious ambition to effect a cure until he has "put his foot into it," and learns by sad experience that which, in vain, the wiser, older heads tried to teach him. For the benefit of my younger *confreres* I shall relate a case from which they may draw their own lessons.

Mrs. R. S., twenty-one years old, newly married, of delicate figure, fair complexion, and blue eyes, very nervous temperament, consulted me on the 25th of October, 1883, complaining of the following symptoms: At the commencement of each menstruation, terrible tormina, which cause her to take to the bed for one, sometimes two days. Menses are regular as to time, but profuse, clotty, and badly smelling, and last a whole week. Weak, empty feeling in stomach, which necessitates eating between meals. Occasionally morning headache. ℞ Sulphur $^{cm}$ (Swan), one dose, dry on tongue.

I did not hear from her until the 16th of May, 1884, when she complained of a dry eruption on hands, especially between thumb and forefinger, which looks like scabies. This eczema came on three weeks ago, commencing on the left hand and then on the right. It itches more when she puts the hands in hot water. Hands and feet are cold. Headache and dizziness for the last two days. Her dysmenorrhœa grew better every month, and the last period came on *without any pains*. Could anybody ask more of a single dose of Sulphur $^{cm}$? Thinking its action to be now exhausted, and that the eruption called for another dose, I gave her one powder of Sulphur $^{cm}$ dry on the tongue.

June 13th, 1884.—Hands got all right very soon, but she had the old pains again with her last menstruation.

I shall not follow up this case any further, suffice it to state that she remains about the same up to this time.

The blunder which had been committed was twofold; not only had the action of the first dose of Sulphur been killed by the too hasty repetition, but also another teaching of Hahnemann and his true disciples had been sinned against, viz.: "If the disease changes its platform, coming from the interior body to its surface, we should consider this as a sure sign of improvement, and leave well enough alone."

Profiting by this mistake, I succeeded better in another case, which may find its place here, inasmuch as it shows the immense long duration of the action of the curative remedy, and teaches, likewise, the difference of a palliative and a curative remedy.

On the 8th of August, 1880, Mr. A. W., forty years old, blonde, blue eyes, and of phlegmatic temperament, a farmer, called at my office complaining as follows:

Since several days a pressive pain in epigastrium going toward the back. No appetite, dry mouth in the morning, tongue covered with a white, slimy coating. Heat in the afternoon without thirst, with occasional chills, so that he prefers to be near the stove or in the sun. Urine burns slightly. ℞ Pulsatilla $^{2c}$, twelve powders; one every two hours.

21st of July, 1881.—He sends for the same medicine that helped him last summer; he complains of the same pain in stomach. ℞ Pulsatilla $^{2c}$, twelve powders, one every two hours.

19th of October, 1882.—Painful numbness of the right arm and hand, especially at night; has to move the arm from place to place. Better through the day during active exercises. ℞ Rhus tox.$^{2c}$, eight powders, night and morning.

19th of May, 1883.—During a warm day he had changed his warm winter clothes to lighter ones, and since then he feels tired and an aching in all his limbs. Amelioration from lying down quietly. Frontal headache worse from motion; roaring in the head and ears, worse from sitting or lying down. Heat and chills interchange; thirsty for much at a time; no appetite. He moved his bowels by drinking some medicinal tea. Pressure in epigastrium, which is also sore to touch. ℞ Bryonia $^{cm}$ (Swan), one dose dry on tongue. Placebo.

24th of May, 1883.—Frequent diarrhœa; movements are scanty and contain indigested food. Tenesmus and passage of flatus during and tenesmus after defication. He has to hurry when he feels the inclination for stools, rolling and rumbling in bowels. Head feels dull, thirst is better, fever is gone, but the pressive pain in epigastrium remains. His mouth is sore, and although he has no appetite, yet his stomach feels so empty, as if he ought to eat something. ℞ Sulphur $^{cm}$ (Swan), one dose dry on the tongue.

28th of August, 1884.—A week ago a furuncle made its appearance on the left shin, and recently a second one. Pain drawing up into left inguinal glands. Slight chilliness toward evening. When he was a child he used to be troubled a great deal with such boils. ℞ Sacch. lact., internally and externally, mutton tallow.

10th of December, 1884.—Furuncles soon got well; has not complained of anything since.

This case furnishes also an illustration to Hahnemann's experience, which he had in the treatment of chronic diseases before he was aware of the power of antipsorics. He found that patients would get better under the then proven remedies as given in his *Materia Medica Pura,* but the improvement would not last. Another remedy would help again, but again a relapse would take place under another form, and so on, until the action of remedies would cease altogether and the patient be doomed. This led him to the great discovery of the anti-sporica and the nature of chronic disease, which will stand as an eternal monument of the grandeur of his genius.

